# Supplementary material for: The virulence regulator CovR boosts CRISPR-Cas9 immunity in Group B Streptococcus
Source: Nat Commun. 2025 Jul 1;16:5678. doi: 10.1038/s41467-025-60871-6 (PMC12216829; doi:10.1038/s41467-025-60871-6)
Supplement: Supplementary file 1 — Supplementary Information [file 41467_2025_60871_MOESM1_ESM.pdf]

Supplementary Figure S1: Pooled CRISPR immunity controls

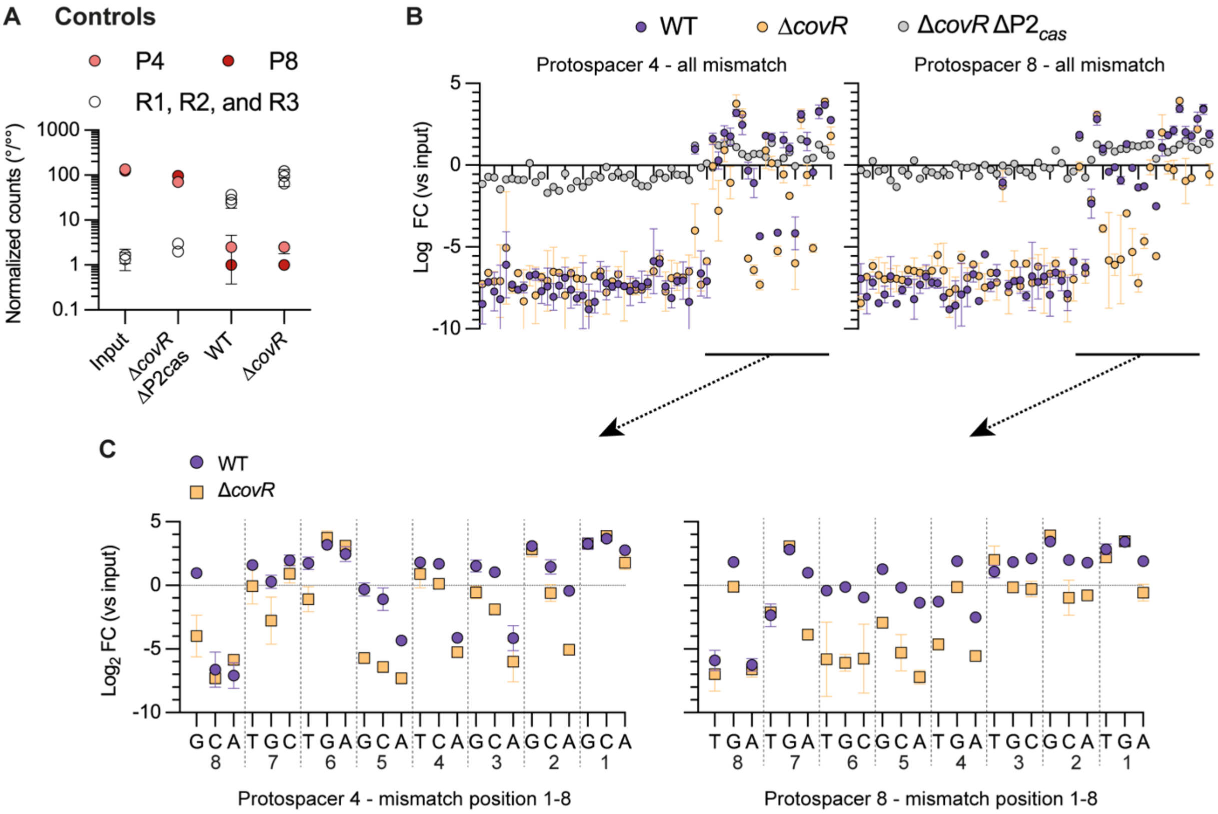

(A) Negative and positive controls for immune selection. The input pool contains protospacers identical to the P4 and P8 wild-type spacers (positive controls; pink and red dots, respectively) and the three R1, R2, and R3 protospacers with random sequences (negative controls; white dots). Proportion of each protospacer is normalized per thousand sequence counts after Illumina sequencing of the input pool and of the ouput pools recovered after transformation of the WT, Δ*covR* and Δ*covR* ΔP2$_{cas}$ mutants. Positive controls are depleted after transformation in the WT and Δ*covR* mutant (immunity), while negative controls are enriched (no immunity). Controls are similarly abundant between the input and Δ*covR* ΔP2$_{cas}$ output pool (absence of Cas9 expression). Each dot represents the mean +/- SD of biological replicate for the WT and Δ*covR* mutant (n = 2), of a single experiment for Δ*covR* ΔP2$_{cas}$ (n = 1), and of technical replicate (n = 3 plasmid purification) for the input pool.
(B) Selected PAM-proximal mutations are depleted in the Δ*covR* mutant. Depletion ratio (Log_2 fold change) of the P4 and P8 protospacers with each possible single mutation at position 1 to 20 (20 x G/A/T/C, excluding WT sequence) in the WT (blue dots), Δ*covR* mutant (orange dots), and Δ*covR* ΔP2$_{cas}$ mutant (grey dots) against the input pool. Each dot represents the mean +/- SD in the same experimental condition as in panel A.
(C) Zoom in on panel B highlighting mutations at position 1 to 8.
Source data is provided in Supplementary Table S1.

Supplementary Figure S2: CovS-dependent phosphorylation contributes to CRISPR-Cas9 immunity.

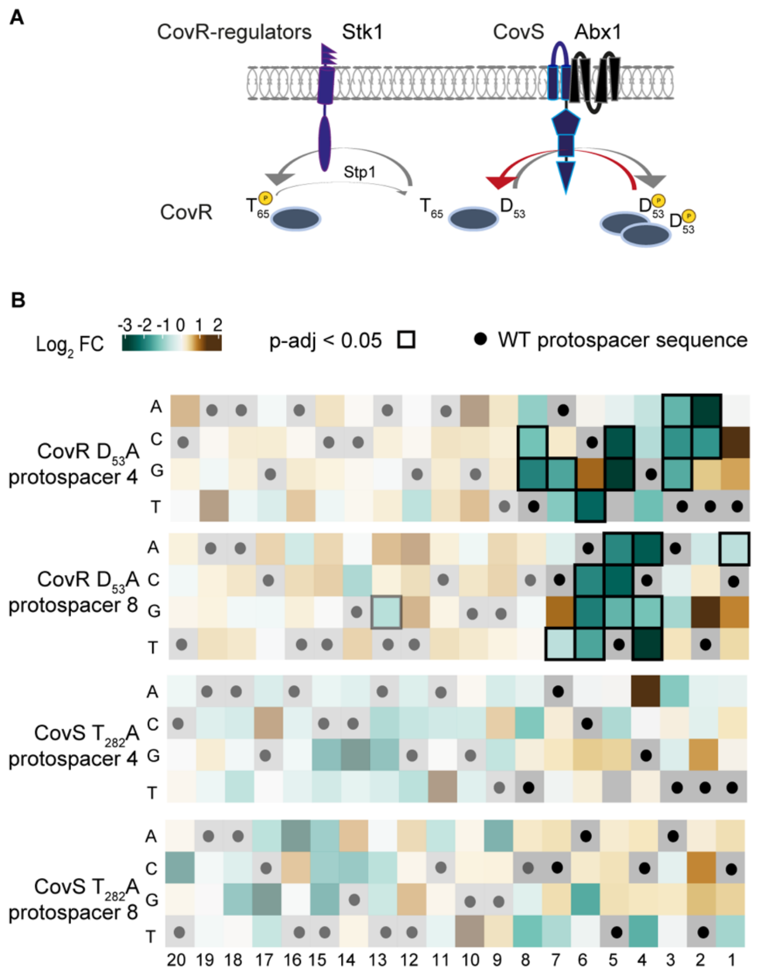

(A) Schematic representation of CovR regulation. In contrast to most two-component systems, CovR is a transcriptional repressor. The membrane-embedded histidine kinase CovS phosphorylates CovR at the D_{53} residue. Activated CovR dimerizes and binds to target promoters to repress gene transcription. The phosphatase activity of CovS inactivates CovR, relieving transcriptional repression. CovS phosphatase activity is itself dependent on the interaction between CovS and the Abx1 protein through their transmembrane domains. Phosphorylation of D_{53} by CovS is antagonistic to phosphorylation of the T_{65} residue by the serine-threonine kinase Stk1, and T_{65} dephosphorylation depends on the cognate phosphatase Stp1. Depending on promoter architecture, full transcriptional derepression may require T_{65} phosphorylation.

(B) CovS-dependent phosphorylation contributes to CRISPR immunity. Bulk immunity assays in biological duplicate (n = 2) were performed with all possible single mutations in the P4 and P8 protospacers using the phosphoablative CovR D_{53}A mutant and the CovS T_{282}A mutant, in which the phosphatase activity of CovS is specifically abolished, leading to hyperphosphorylation of the D_{53} residue of CovR. The gain in immunity for all possible single mutations in the P4 and P8 protospacers was calculated relative to the wild-type (WT) strain (Log2 fold change: green to brown). The wild-type spacer sequences are depicted with a dot at each position. Statistical analysis with DESeq2 uses the Wald test to compute p-values, followed by Benjamini-Hochberg correction to adjust for multiple comparisons. Significances (p-adj < 0.05) are highlighted by black square borders. Raw count, p-values, and p-adj are provided in Supplementary Table S4.
