# Supplementary material for: The virulence regulator CovR boosts CRISPR-Cas9 immunity in Group B Streptococcus
Source: Nat Commun. 2025 Jul 1;16:5678. doi: 10.1038/s41467-025-60871-6 (PMC12216829; doi:10.1038/s41467-025-60871-6)
Supplement: Supplementary file 2 — Description of Additional Supplementary Files [file 41467_2025_60871_MOESM2_ESM.pdf]

Description of Additional Supplementary Files:

Supplementary Data 1: Bulk CRISPR immunity assay in BM110 included 3 sheets. Supplementary Data S1A: List of protospacer sequences with single mismatch. Supplementary Data S1B: Protospacer absolute counts (2FAST2Q analysis). Supplementary Data S1C: Increased immune efficiency in ΔcovR mutant (DESeq2 analysis).

Supplementary Data 2: RNA-seq analysis of ΔcovR mutants included 3 sheets. Supplementary Data S2A : RNA-seq analysis of ΔcovR mutant in NEM316. Supplementary Data S2B : RNA-seq analysis of ΔcovR mutant in 2603V/R. Supplementary Data S2C : RNA-seq analysis of ΔcovR mutant in A909.

Supplementary Data 3: oligonucleotides

Supplementary Data 4: Bulk CRISPR immunity assay in CovRD53A and CovST282A mutants included 3 sheets. Supplementary Data S4A: Protospacer absolute counts (2FAST2Q analysis). Supplementary Data S4B: Immune efficiency in CovRD53A mutant (DESeq2 analysis). Supplementary Data S4C: Immune efficiency in CovST282A mutant (DESeq2 analysis).
